# Environment and Gene Association With Obesity and Their Impact on Neurodegenerative and Neurodevelopmental Diseases

**DOI:** 10.3389/fnins.2020.00863

**Published:** 2020-08-28

**Authors:** María Teresa Flores-Dorantes, Yael Efren Díaz-López, Ruth Gutiérrez-Aguilar

**Affiliations:** ^1^Laboratorio de Biología Molecular y Farmacogenómica, Centro de Investigación de Ciencia y Tecnología Aplicada de Tabasco, División Académica de Ciencias Básicas, Universidad Juárez Autónoma de Tabasco, Villahermosa, Mexico; ^2^Laboratorio de Enfermedades Metabólicas: Obesidad y Diabetes, Hospital Infantil de México “Federico Gómez,” Mexico City, Mexico; ^3^División de Investigación, Facultad de Medicina, Universidad Nacional Autónoma de México (UNAM), Mexico City, Mexico

**Keywords:** obesity, neurodegenerative diseases, neurodevelopmental diseases, environment, genes

## Abstract

Obesity is a multifactorial disease in which environmental conditions and several genes play an important role in the development of this disease. Obesity is associated with neurodegenerative diseases (Alzheimer, Parkinson, and Huntington diseases) and with neurodevelopmental diseases (autism disorder, schizophrenia, and fragile X syndrome). Some of the environmental conditions that lead to obesity are physical activity, alcohol consumption, socioeconomic status, parent feeding behavior, and diet. Interestingly, some of these environmental conditions are shared with neurodegenerative and neurodevelopmental diseases. Obesity impairs neurodevelopment abilities as memory and fine-motor skills. Moreover, maternal obesity affects the cognitive function and mental health of the offspring. The common biological mechanisms involved in obesity and neurodegenerative/neurodevelopmental diseases are insulin resistance, pro-inflammatory cytokines, and oxidative damage, among others, leading to impaired brain development or cell death. Obesogenic environmental conditions are not the only factors that influence neurodegenerative and neurodevelopmental diseases. In fact, several genes implicated in the leptin–melanocortin pathway (*LEP*, *LEPR*, *POMC*, *BDNF*, *MC4R*, *PCSK1*, *SIM1*, *BDNF*, *TrkB*, etc.) are associated with obesity and neurodegenerative and neurodevelopmental diseases. Moreover, in the last decades, the discovery of new genes associated with obesity (*FTO, NRXN3, NPC1, NEGR1, MTCH2, GNPDA2*, among others) and with neurodegenerative or neurodevelopmental diseases (*APOE, CD38, SIRT1, TNF*α, *PAI-1, TREM2, SYT4, FMR1, TET3*, among others) had opened new pathways to comprehend the common mechanisms involved in these diseases. In conclusion, the obesogenic environmental conditions, the genes, and the interaction gene–environment would lead to a better understanding of the etiology of these diseases.

## Introduction

Obesity is an excess of fat body mass that may decrement health. Obesity is caused by an energy imbalance due to an excess of food intake and less physical activity. The World Health Organization (WHO) has reported that worldwide obesity prevalence has tripled since 1975. In 2016, the prevalence of overweight and obesity was over 1.9 billion adults, 39% overweight, and 13% obese (World Health Organization [WHO], 2020). Body mass index (BMI), given by dividing the weight by height (kg/m^2^), is used for classifying people as obese (BMI above 30 kg/m^2^).

The obesity epidemic has dramatically increased in the last decades, and it goes in parallel with the change in our environment by unhealthy diet (sugar-sweetened beverages, fried foods, etc.), physical inactivity, sedentary lifestyle, and poor sleeping (Hruby et al., 2016).

In addition, genetics is also implicated in the development of obesity, explaining over 40% of the heritability of this disease. Several studies have demonstrated that genes in the melanocortin system control the energy balance [reviewed in Xu et al. (2011) and Hill and Faulkner (2017)]. Mutations in these genes, such as melanocortin 4 receptor (MC4R), have been associated with monogenic obesity (Farooqi et al., 2000). However, studies about common or polygenic obesity have revealed over 900 genetic variants associated with obesity by the approach of genome-wide association studies (GWAS) (Locke et al., 2015; Yengo et al., 2018).

Moreover, obesity is a risk factor to develop several neurological consequences (O’Brien et al., 2017), such as dementia and Alzheimer disease [AD, reviewed in Arnoldussen et al. (2014) and Anjum et al. (2018)]. Obesity is a comorbidity for psychiatric disorders that may influence behavior, cognition, and mood, suggesting that common biological pathways in the central nervous system (CNS) are implicated in obesity and psychiatric disorders (Proulx and Seeley, 2005).

In this journal’s issue, a compendium of papers focused on obesogenic environmental conditions that affect neurodevelopment and neurodegeneration was collected. In this review, we will address aspects, such as obesogenic environment leading to develop obesity. In addition, the principal genes that have been associated with obesity and that are implicated in neurodegenerative diseases (NDgDs) or neurodevelopmental diseases (NDvDs) as well as the new genes associated with NDgD or NDvD are described.

## Obesogenic Environment

To prove that an obesogenic environment could play an important role in developing obesity, dogs exposed to an obesogenic environment (owned by obese people) presented a higher prevalence of obesity compared to dogs with lean owners (Mason, 1970). Then, obesogenic environmental conditions have been part of the increment on this disease.

Two important components, the environment (nurture) and the genes (nature), are risk factors to develop common obesity. These two components are frequently studied independently; however, the interaction between gene and environment can increase the susceptibility to develop the disease. The genes are part of nature and have been defined as the nucleotides that form our DNA (deoxyribonucleic acid). DNA differs in some base pairs among individuals, giving genetic variation and individual differences in a trait (Wood, 2019). However, environment is part of nurture; that is, a non-genetic thing that could modify a trait (Wood, 2019).

The United Kingdom Biobank (UK Biobank) has collected data of close to half a million United Kingdom citizens of middle to old age about lifestyle factors and genetics (Sudlow et al., 2015). More than 130 lifestyle factors, such as socioeconomic status, general health, mental health, sleep, physical activity, alcohol consumption, smoking, diet, the genetic risk score (GRS), among others, were analyzed to study the interaction between gene and environment leading to obesity. Gene–environment interaction is defined as the response of an individual to environmental stimuli based on his genotype. In other words, the genes confer susceptibility, but the environment could influence and modify the genotype (Reddon et al., 2016).

Analysis of the UK Biobank database showed that the most significant environmental factors that could influence the development of obesity, in the presence of genetic risk variants, were physical activity, alcohol consumption, and socioeconomic status (Sudlow et al., 2015; Rask-Andersen et al., 2017). Moreover, parent feeding behavior and diet had also been implicated in the development of obesity (Dalle Molle et al., 2017).

### Physical Activity

When it comes to obesogenic environment, we need to think about the main arms of the energy balance, which are energy expenditure and energy intake. With respect to lower energy expenditure, a sedentary lifestyle, or physical inactivity, due to prolonged watching TV hours, interacts with the genetic predisposition causing the development of obesity (Qi et al., 2012b). A study analyzing 20,000 men demonstrated that having a physically active lifestyle reduces 40% of the genetic predisposition for obesity by analyzing 12 variants associated with obesity (Li et al., 2010). A meta-analysis of more than 110,000 individuals supported the previous results, indicating that the physical activity counterbalances the genetic predisposition to develop obesity (Ahmad et al., 2013).

### Alcohol Consumption

Alcohol consumption was another gene–environment associated with BMI (Rask-Andersen et al., 2017). In fact, alcohol consumption might reduce the effect of the obesity genetic variants by reducing the BMI. This is consistent with data showing that alcoholic patients present lower physical activity (Liangpunsakul et al., 2010) and a lower BMI (Addolorato, 2000) as a consequence of an increase in lipolysis and disorders of lipid metabolism (Steiner and Lang, 2017). In fact, it has been demonstrated that chronic alcohol consumption could provoke lipodystrophy in rats, triggered by a disturbance on lipogenesis (Zhang et al., 2015). A higher lipolysis and fatty acid release will provoke the transportation of the fatty acids to the liver, leading to their accumulation and provoking hepatic steatosis over time (Kema et al., 2015).

### Socioeconomic Status

Several studies have analyzed the relationship between socioeconomic status and obesity. It has been reported that obesity prevalence increases with higher deprivation levels (National Obesity Observatory, 2012) due to a worse diet (Darmon and Drewnowski, 2008) and less physical activity (Giles-Corti, 2002). Interestingly, in rich countries, the obesity prevalence was higher among poor people and did not change among the wealthiest. However, in poor countries, obesity and overweight is higher in the upper class (Templin et al., 2019). In developing countries, such as Mexico, a new middle class (poor people who became wealthier) has the most risk to become obese (Levasseur, 2015).

To address how education could influence BMI, a study with siblings sharing the same environment was performed. This study demonstrated that higher education levels correlate with a lower BMI (Kim, 2016). Educational level could affect the selection and purchase of healthy food due to the knowledge of healthy food (Bhurosy and Jeewon, 2014). Another study performed in children demonstrated that severe obesity increased with a lower household head education and lower urbanization level (Ogden et al., 2018).

Moreover, many “environmental layers” could affect obesity predisposition, such as intrauterine environment, mother–child interaction, food/community environment, parent feeding behavior, among others, representing the biological or social influence for any person to develop the disease (Dalle Molle et al., 2017). Of these layers, we will briefly describe parent feeding behavior and food (diet) as part of the energy intake, an important arm of the energy balance.

### Parent Feeding Behavior

One of the environmental layers influencing child appetite is parent feeding behavior. For example, giving food as a reward of good behavior is associated with a higher intake of unhealthy foods and beverages (Dalle Molle et al., 2017).

A poor eating self-regulation in children has been associated with higher body weight (Hughes and Frazier-Wood, 2016). Picky eating is common in preschool children, which leads to parental anxiety and family conflicts (Kumar et al., 2018). Authoritative parenting has a positive correlation with non-picky eating in their toddler, suggesting that this parent feeding behavior could overcome the feeding difficulties (Podlesak et al., 2017). Lower weight has been predicted with mothers who promote positive child body image; however, feeding practices of pushing to eat have been associated with weight gain (Damiano et al., 2016). Then, nurture plays an important role in child eating behaviors and gene–environment interaction to shape child appetitive traits (Wood, 2019).

### Diet

Diet is one of the main environmental factors that influence obesity development. An accelerated lifestyle has changed dietary habits, inciting people to look for ready-to-go food, instead of preparing their food at home. In fact, a United States National Nutritional Survey (from 1965 to 2008) reported that home-consumed diet has decreased 23% and most of the Americans have their calorie intake from processed foods purchased in restaurants or grocery stores (Smith et al., 2013). A study in the United Kingdom demonstrated that consumption of out-of-home (restaurants, cafes, and takeaways) meals has a higher energy intake (75–104 kcal per day) compared to those of people who rarely consume them (Goffe et al., 2017). On the other hand, frequent cooking at home has been associated with better healthy eating index, regardless low or high income (Wolfson et al., 2020).

Epidemiological studies have shown that in the period of 2005–2010, 14% of American young adults obtained their caloric needs from sugar-sweetened beverages (Ervin and Ogden, 2013). In Mexico, a country with one of the world’s highest obesity prevalence, 17.5% of children and 19% of the adults obtained their daily caloric intake from sweetened beverages (Stern et al., 2014).

A systematic review of 32 studies reported a positive association between sugar-sweetened beverage consumption and the obesity risk (Bucher Della Torre et al., 2016). Moreover, a meta-analysis showed that consumption of soft drinks has increased in the last decades, and 75% of beverages and food contained added sugar. Then, drinking sugar-sweetened beverages increases the risk of obesity, diabetes, and metabolic syndrome (Bray and Popkin, 2014). In addition, obesity-genetic predisposition is associated with the positive correlation between soft drink and fried food consumption and adiposity traits (Qi et al., 2014; Olsen et al., 2016). A review suggested that eating fried foods at least four times per week gives a higher risk on developing obesity and other chronic diseases, such as type 2 diabetes (T2D) and hypertension, leading to coronary artery disease (Gadiraju et al., 2015).

Another study showed that eating more fruits and vegetables and avoiding red and processed meats could improve cardiometabolic profiles (Schwedhelm et al., 2019).

For this reason, WHO has published dietary recommendations, suggesting a higher intake of fruits, vegetables, legumes, nuts, and whole grains, limiting to less than 10% of free sugars and 30% from fats of the total intake (World Health Organization [WHO]., 2018). Even though these recommendations have been published, it has been shown that there are several barriers to eat healthy food, such as household income and cooking and eating behaviors (Wolfson et al., 2019).

A recent study demonstrated that diets in different populations are “unhealthy, unsustainable, and unequitable” (Fanzo and Davis, 2019). As abovementioned, people are eating “unhealthy,” with high intake of sugar and fats. The diet is a link between humans and the environment. The production of plants and animals for human consumption is changing the environment, provoking biodiversity loss and becoming “unsustainable.” Finally, socioeconomic status marginalizes some people to access healthy food, the diet becoming “inequitable.”

Therefore, interventions to control obesity have to consider several environmental factors that had been associated with obesity. For example, in Latin America, some of the intervention programs have implemented elevated taxes for sugar-based foods, marketing control, and consumer education to select healthier food (Popkin and Reardon, 2018). The global network, International Network for Food and Obesity Monitoring and Action Support (INFORMAS), implemented in Mexico nine different actions to reduce obesity: provision and promotion of healthy food, increase the offer of healthy food, a comprehensive package, combat obesity using the money obtained from the taxes over the sugar-sweetened beverages, among others (Nieto et al., 2019).

As addressed above, environment has many different edges, so it cannot be simply studied. For example, using the new technologies such as sequencing, it could take decades, trying to sequence the whole environment. Therefore, it has been an easier option to sequence the human genome than the environment (Bogardus and Swinburn, 2017). Sequencing the human genome will help to understand how genetic mutations could provoke a disease (monogenetic) or how different genetic variants give susceptibility to develop the disease (polygenetic).

## Genetics of Obesity

When we talk about genetics, we have to define heritability as “the proportion of observed variation in a particular trait that can be attributed to inherited genetic factors” (Merriam-Webster Dictionary, 2020). A systematic review of obesity heritability, based on the BMI of twins studies (140,525 twins) and family studies (42,968 family members), revealed that BMI heritability ranged from 47 to 90%. In addition, they demonstrated that the genetic contribution to BMI was higher during childhood than adulthood (Elks et al., 2012).

Obesity is classified in three different ways: monogenetic, polygenetic, or syndromic obesity. In this review, we will only focus on explaining monogenetic and polygenetic obesity.

### Monogenic Obesity

Monogenetic obesity is caused by mutations (not common changes in the DNA) of one gene, typically causing severe early-onset obesity. This type of obesity is rare, and approximately 7.3% of the severe early-onset obesity is affected in one gene (Kleinendorst et al., 2018). Some of the genes associated with monogenetic severe obesity are: leptin (*LEP*), leptin receptor (*LEPR*), proopiomelanocortin (*POMC*), *MC4R*, preproconvertase 1 (*PCSK1*), single minded 1 (*SIM1*), brain-derived neurotrophic factor (*BDNF*), and tyrosine kinase receptor tropomycin-related kinase B (*TrkB*) (Ramachandrappa and Farooqi, 2011).

Most of the proteins involved in the monogenic obesity are involved in the leptin–melanocortin signaling pathway. This pathway is important in controlling the energy balance in the hypothalamus by coordinating the energy intake and the energy expenditure (Morton et al., 2014; van der Klaauw and Farooqi, 2015). Leptin stimulates neurons expressing POMC, producing melanocortin peptides that will bind the MC4R controlling the energy balance. Mutations in *POMC* and *MC4R* genes lead to severe obesity due to hyperphagia (Krude et al., 1998; Farooqi et al., 2003). Mutations in *MC4R* are the most common in monogenetic obesity, present in more than 5% of childhood obesity (Farooqi et al., 2000; Vaisse et al., 2000). Mutations on *PCSK1* lead to a misprocessing of melanocortin peptides, then leading to obesity and abnormalities of glucose homeostasis and adrenal function (O’Rahilly et al., 1995; Jackson et al., 1997). The transcription factor SIM1 causes severe obesity due to hyperphagia and reduces the paraventricular nucleus (PVN) of the hypothalamus (Michaud, 2001; Ramachandrappa et al., 2013). *BDNF* is also expressed in the hypothalamus, and it is downstream MC4R signaling promoting anorectic signal and locomotor activity (Kernie, 2000; Lebrun et al., 2006). Mutations in *BDNF* and its receptor, *TrkB*, are associated with obesity and hyperphagia either in humans or mice (Xu et al., 2003; Yeo et al., 2004).

Details of the function of these genes will be presented in the section *Monogenetic Obesity Genes*.

### Polygenetic Obesity

Common obesity is characterized to be associated with several variants or polymorphism of different genes, polygenetic. These polymorphisms or single nucleotide variants (SNVs) are changes of one nucleotide of the DNA sequence and are common variants in the population.

Fifteen years ago, technology had a great impact on genetics by creating new molecular biology methods able to analyze several variants at the same time. In addition, the formation of big consortia, such as the UK Biobank or Genetic Investigation of ANthropometric Traits (GIANT) consortium, helped increase the sample size for epidemiological or genetic studies.

The GWAS were created based on analyzing thousands or millions of variants (covering more than 75% of the human genome) at the same time in thousands of individuals (case-control cohorts). The GWAS are hypothesis-free studies, meaning that we ignore the biological effects of the variants analyzed.

In 2007, F*TO* was the first gene associated with obesity discovered by the GWAS approach (Frayling, 2007). Then in 2009, the first three obesity GWAS were published, reporting 16 *loci* associated with obesity (Meyre et al., 2009; Thorleifsson et al., 2009; Willer et al., 2009). Since then, several meta-analyses had identified different variants associated with obesity in different populations (Locke et al., 2015; Yengo et al., 2018) or with obesity-related traits, such as body fat distribution (Pulit et al., 2019) in more than 300,000 or 700,000 individuals. Now, 941 near-independent variants have been associated with BMI, and 346 loci were associated with body fat distribution (Yengo et al., 2018; Pulit et al., 2019).

Interestingly, many of the new genes associated with obesity were found to be involved in neurogenesis, in the development of the CNS, in pathways such as appetite and food intake regulation (Locke et al., 2015; Yengo et al., 2018). In addition, a human compendium of 578 genes associated with body weight and food intake and expressed in the brain was published by collecting data from several GWAS and some candidate genes (Ignatieva et al., 2016).

Some of the genes associated with obesity discovered by GWAS approach are *MC4R, POMC, FTO, NRXN3, NPC1, NEGR1, GNPDA2, MTCH2, ETV5*, among others. Some of them are related to synaptic function and neurotransmitter signaling (*NEGR1*, *NRXN3*, *CADM2*, *GRID1*, *ELAVL4*, and *SCG3*) and energy homeostasis (*POMC*, *MC4R*, *BDNF*, *ETV5*, *HNF4G*, and *TLR4*) (Locke et al., 2015).

The GWAS opened a new gate to understand the biological pathways involved in obesity. This hypothesis-free approach reveals new variants that are associated with obesity, without knowing their target gene and the effect that could have over it. Then, the challenge now is to go from genomics to physiology, describing the function of the new genes associated with obesity (Gutierrez-Aguilar et al., 2012, 2014).

Polygenic variants have a modest effect. Then, GRS was created to represent “the number of risk variants across all the genome,” meaning that the higher the GRS, the higher susceptibility to develop obesity (Loos and Janssens, 2017). Several efforts have been performed to obtain an algorithm that could predict the risk for developing obesity (Loos and Janssens, 2017; Khera et al., 2019). Finally, Khera et al. (2019) published a genome-wide polygenic score that integrates all available common variants for obesity into a quantitative measure of inherited susceptibility. This score is able to predict the risk of developing obesity, as well as differences in weight during childhood (Khera et al., 2019).

In the last 15 years of the genomic era, hundreds of variants have been associated with obesity. Ultimately, obesity genetic risk prediction could be used to know the individual’s genetic susceptibility to develop the disease. Therefore, as a principle of precision medicine, strategies and treatments could be personalized for each individual in the future, known as pharmacogenomics. However, biology is now the missing piece for solving the obesity puzzle. Many years may take to be able to understand the effect of each of the variants associated with obesity and to understand their function and their influence in the biology of obesity.

More details of the function of these genes will be presented in section *Polygenetic Obesity Genes*.

## Gene–Environment Interaction Leading to Obesity

Many studies have reported that the predisposition to develop obesity is by the influence of either the environment or gene variants. In this section, we will discuss individual variants or the GRS (adding the effect of several gene variants associated with obesity) and their association with some obesity traits, as well as some environmental factors.

Individual variants are studied to simplify the understanding of the interaction between gene–environment. An example of this is the interaction of two well-known obesity variants (*FTO* rs9939609 and *MC4R* rs17782313) with lifestyle measured. The study demonstrated that changes in lifestyle by implementing physical activity and adherence to a Mediterranean diet can modulate the obesity risk conferred by *FTO* and *MC4R* variants (Corella et al., 2012). In addition, gene variants could have an effect on weight loss depending on the diet. *FTO* variant is associated with change in appetite or abdominal fat distribution when exposed to high or low protein diet (Zhang X. et al., 2012; Huang et al., 2014).

The BMI heritability in twin and family studies is 47–90% (Elks et al., 2012). However, an obesity GWAS meta-analysis, analyzing 941 gene variants, explained that those variants represented only [∼6% of the variance of BMI (Yengo et al., 2018)], suggesting that all those variants associated with obesity have modest effects. Then, where is the rest of the heritability? The answer to this question has been called the “missing heritability” that could partly be explained by the influence of the environment over the genes. Several reviews have summarized the state of the art of the interaction between genes and environment in obesity, eating behavior, and diet, among others (Dalle Molle et al., 2017; Heianza and Qi, 2017; Heianza and Qi, 2019; Li and Qi, 2019).

Genetic risk score takes into account several genetic variants that could explain a trait. For example, some of the lifestyle factors that may modify the obesity GRS are sugar-sweetened beverages, where over 30 obesity gene variants were analyzed, and demonstrated interaction between genes and environment (Qi et al., 2012a; Brunkwall et al., 2016). As mentioned above, the UK Biobank data analysis demonstrated the relationship between genetic risk and environment (Sudlow et al., 2015; Rask-Andersen et al., 2017; Tyrrell et al., 2017; Nagpal et al., 2018). For example, the analysis of the GRS of 94 genetic variants were associated with physical activity, sedentary lifestyle, and socioeconomic status (Rask-Andersen et al., 2017).

Therefore, our genes confer susceptibility to develop obesity; however, we can modify the environment to reduce the risk. Then, improving adherence to healthy dietary patterns had a benefit, even in the presence of an obesity genetic risk (Wang et al., 2018c).

Then, gene–environment interactions are explaining part of the missing heritability; however, more research is needed to better understand how the genes give susceptibility to develop a disease in the presence of an environment that enhances the trait. Interestingly, some of the environmental factors associated with obesity are also risk factors to develop NDgD or NDvD, as described below.

## Neurodegenerative and Neurodevelopmental Diseases

Neurodegenerative diseases and NDvDs are multifactorial disorders where environment and genetics play an important role for their development (Cardoso et al., 2019; Dunn et al., 2019).

Neuronal loss leads to neurodegeneration due to accumulation of abnormal proteins in the brain. Dementia could be described as the impairment of cognitive function, and the most common NDgDs are AD (60–70% of the cases) and Parkinson’s disease (PD).

Alzheimer disease is characterized by an abnormal accumulation and deposition of β-amyloid peptide on the amyloid plaque (Haass and Selkoe, 2007). In addition, TAU protein is hyperphosphorylated, producing paired helical filaments integrated in neurofibrillary tangles (Zenaro et al., 2017). On the other hand, PD is characterized by postural instability, resting tremor, stiffness, bradykinesia, caused by a progressive loss of dopaminergic neurons, and aggregates of α-synuclein (Poewe et al., 2017). Another NDgD is Huntington’s disease (HD) characterized by progressive brain disorder leading to movement, loss of cognitive conditions, emotional problems, and psychiatric symptoms (Snowden, 2017).

It has been demonstrated that obesity is a risk factor for mild cognitive impairment, independent of age (Elias et al., 2005; Hassing et al., 2010). Moreover, a meta-analysis reported the association between obesity and neurological disorders, demonstrating that obesity doubles the risk for AD (Anstey et al., 2011), and diabetes also confers risk (Profenno et al., 2010). Metabolic syndrome (obesity, T2D, hypertension, hypercholesterolemia, and hypertriglyceridemia) constitutes a risk factor for developing PD (Nam et al., 2018).

On the other hand, NDvDs are a group of conditions that affect the development of the nervous system, leading to learning disability, affecting self-control, emotions, and memory, as well as impairment in personal, occupational, and social functioning (Maher et al., 2017). Obesity has also been associated with neurodevelopmental disorders, such as autism (Criado et al., 2018) and schizophrenia (SCZ) (An et al., 2018).

Several authors have already reviewed the implications of obesity, and its metabolic dysfunctions could contribute to neurological consequences (O’Brien et al., 2017), as well as the impact on NDgD (Mazon et al., 2017). Briefly, overweight and obesity provoke metabolic changes that damage the CNS by altering synaptic plasticity and leading to neural death by either cell necrosis or apoptosis (Mazon et al., 2017). More details will be explained in Section “*Common Biological Mechanisms Between Obesity and Neurodegenerative and Neurodevelopmental Diseases*.” It is evident that obesity provokes metabolic and physiological changes that lead to the development of NDgD or NDvD.

## Environmental Factors Influencing Neurodegenerative or Neurodevelopmental Diseases

Some of the environmental factors that have been implicated in a cognitive decline, dementia, or NDgD are physical activity, diet, and stress (lack of sleep) (Zhao et al., 2018; Gubert et al., 2020). In particular, for PD, lifestyle factors such as heavy alcohol consumption and cigarette were associated with the risk of developing the disease (Paul et al., 2019). Moreover, other illicit substances, heavy metals (iron, copper, manganese, lead, and mercury), pesticides, are other environmental factors that could provoke PD (Ball et al., 2019).

It has been described that obesity could lead to poorer neurodevelopment abilities, such as attention, inhibitory control, working memory, problem-solving, and fine motor skills (Mina et al., 2017). These abilities are impaired in NDvDs such as autism spectrum disorders (ASD), attention-deficit hyperactivity disorder (ADHD), and diseases associated with obesity (Manu et al., 2015; Criado et al., 2018; Hanć and Cortese, 2018).

A lower intelligence quotient score in childhood is associated with weight gain and obesity in later adulthood (Chandola et al., 2006; Yu et al., 2010). Moreover, midlife obesity is associated with lower cognitive ability, memory, verbal ability, and spatial abilities in late life (Dahl et al., 2010; Hassing et al., 2010). During early-life years, exposure to different factors such as education level, food deficiency, learning ability, family-related factors could increase the risk to develop cognitive impairment and dementia in advance age (Wang X. J. et al., 2019).

Maternal obesity, an environmental factor for the offspring, affects the cognitive function and mental health of the offspring. Maternal obesity could influence memory, learning, and some NDvDs, such as ADHD and ASD, as well as NDgD (Contu and Hawkes, 2017; Edlow, 2017). In addition, offspring of obese mothers present behavioral abnormalities, decreased sociability, anxiety, and feeding disorders (Edlow, 2017). In fact, maternal obesity increases the risk for intellectual disability in 1.3–3.6-fold, ADHD in 1.6–2.8-fold, and 2-fold in the difficulty of regulating emotions (Edlow, 2017).

Another NDvD is SCZ; its risk factors to develop it include obesity, poor diet, physical activity, genetic vulnerability, stress, environmental toxins, among others (Debnath et al., 2015).

Thus, obesity and NDgD/NDvD share the same environmental risk factors (physical activity, diet, socioeconomic status, stress, among others), suggesting that all these factors could modulate common biological mechanisms, regulating the CNS. In the next section, we address the common biological mechanism shared by obesity and NDgD/NDvD.

Neurodegenerative disease or NDvD development is influenced by exposure to environmental factors and by the genetic background of each person. Some of the genes associated with NDgD or NDvD are described in Section “Genes Associated With Neurodegenerative and Neurodevelopmental Diseases.”

## Common Biological Mechanisms Between Obesity and Neurodegenerative and Neurodevelopmental Diseases

Several authors have reviewed the plausible common biological mechanisms between obesity and neurodegeneration/neurodevelopment, which are briefly explained below (Mazon et al., 2017; O’Brien et al., 2017; Pugazhenthi et al., 2017).

Obesity is a consequence of an excessive energy intake, provoking a hypertrophy of the adipose tissue. Consequently, dysfunction and inflammation of the adipose tissue trigger impaired insulin signaling, altered secretion of adipokines and cytokines, compromised triglyceride storage, and liberation of free fatty acids. The high levels of free fatty acids contribute to insulin resistance (IR) (Schneeberger et al., 2014), which has been linked to neurocognitive dysfunction (Stoeckel et al., 2016). IR and chronic hyperglycemia induce oxidative stress and inflammatory responses, provoking neuronal death and impairing cognitive processes (Treviño et al., 2015). Pro-inflammatory adipokines, including interleukin (IL)-1, IL-6, IL-1β, Tumor Necrosis Factor-Alpha (TNFα), plasminogen activator inhibitors (PAI-1), C-reactive protein, and leptin, have been proposed to be part of the neuroinflammation triggering neurodegeneration [reviewed in Arnoldussen et al. (2014)].

Leptin, a hormone secreted by the adipose tissue, reaches the hypothalamus (to control energy balance) and the hippocampus, regions involved in processes such as synaptic plasticity, memory, and cognition (Irving and Harvey, 2014). Besides the pro-inflammatory responses, other common biological features between obesity and neurodegeneration are oxidative damage, energy metabolism failures, and mitochondrial and neurotransmission systems dysfunction, ultimately leading to cell death [reviewed in Mazon et al. (2017)].

As mentioned above, maternal obesity exposes the offspring to inflammatory cytokines, which has been associated with low birth weight and premature birth, but interestingly is also associated with neural development, leading to disorders like SCZ, ADHD, and ASD (Buka et al., 2001; Donev and Thome, 2010; Blackmore et al., 2011; Angelidou et al., 2012). In fact, inflammatory mediators cross the blood–placenta barrier influencing fetal development. Then, maternal obesity produces a raise on inflammatory markers in the hippocampus of the offspring (Sullivan et al., 2015).

In addition, inflammation regulates serotonin function. Interferon alpha-treated rats showed decreased serotonergic axons in the amygdala and the ventral media prefrontal cortex (Ishikawa et al., 2007). Moreover, maternal high-fat diet consumption diminished the serotonin synthesis, leading to anxiety behaviors in the offspring (Sullivan et al., 2010).

Another reason how obesity affects the brain development is the fact that glucose can cross the blood–placenta barrier. However, the maternal insulin does not cross it, leading to insulin production by the baby. This hormone acts as a growth factor, then hyperinsulinemia in the prenatal period might alter brain development (Stachowiak et al., 2013). On the other hand, in ASD children, higher leptin levels were detected compared to healthy children (Ashwood et al., 2008).

## Genes Associated With Obesity and Neurodegenerative and Neurodevelopmental Diseases

As we previously mentioned, many genes are involved in the susceptibility to develop obesity and NDgD/NDvD. However, in the next section, we will describe some of the most important genes associated with these diseases, their function, and the plausible mechanism overlapping among these complex diseases.

### Genes Associated With Obesity and Its Impact on Neurodegenerative or Neurodevelopmental Diseases

Some of the genes that code for the leptin–melanocortin pathway proteins are associated with monogenetic or polygenic obesity.

In this section, we will briefly describe the genes involved in this pathway and that have been associated with monogenic obesity, as well as their effect in developing NDgD and NDvD (Table 1). These genes are *LEP*, *LEPR*, *POMC*, *CAR*T, *NPY*, *MC4R*, *PCSK1*, *SIM1*, *BDNF*, and *TrKB*.

**TABLE 1:** Genes associated with obesity and NDgD or NDvD.

GENE	NDgD	NDvD	REFERENCES
**Monogenic**			
*LEP*	PD		Polymeropoulos, 1997; Singleton, 2003
*LEPR*	PD	Cognitive damage	Platt et al., 2016; Cordner et al., 2019
*POMC*	AD, HD		van der Burg et al., 2008; Do et al., 2018
*CART*	HD, PD		Lin et al., 2018; Cheong et al., 2019
*NPY*	AD, PD, HD		Ahmed et al., 2019; Li et al., 2019
*MC4R*	AD		Giuliani et al., 2011; Do et al., 2018
*PCSK1*	AD	PWS	Oddo et al., 2003; Hokama et al., 2014; Castillo et al., 2017; Ramos-Molina et al., 2018
*SIM1*	PD	SCZ	Osterberg et al., 2011; Purcell et al., 2014
*BDNF*	AD	Depressive disorder	[Reviewed in Balietti et al. (2018) and Zaw and Taneepanichskul (2019)]
*TrkB*	PD		[Reviewed in Jin (2020)]
**Polygenic**			
*FTO*	AD		Vagelatos and Eslick, 2013; Li et al., 2018
*NRXN3*	AD	ASD	Vaags et al., 2012; Zheng et al., 2018; Hishimoto et al., 2019
*NPC1*	AD	SCZ	Rouillard et al., 2016; Kawazoe et al., 2018
*NEGR1*	AD	SCZ, ASD	Karis et al., 2018; Ni et al., 2018; Szczurkowska et al., 2018; Raghavan et al., 2019
*MTCH2*	AD	SCZ	Purcell et al., 2014; Karch et al., 2016
*GNPDA2*	PD		Lachén-Montes et al., 2019
*APOE*	AD		Lambert et al., 2013; Liu et al., 2013; Shi et al., 2017; Wray et al., 2018
*CD38*	PD		Saad et al., 2011; Chang et al., 2018
*SIRT1*	AD, PD	SCZ	Kishi et al., 2011; Zhang A. et al., 2012; Rana et al., 2019
*TNF*α	AD, PD	SCZ, ASD	Baj and Seth, 2018; Bodnar et al., 2018
*PAI1*	PD		Pan et al., 2018
*TREM2*	AD		Yeh et al., 2017
*SYT4*	AD		Zhang et al., 2009
*FMR1*		FXS	Tassone et al., 2000, 2007; Li et al., 2020
*TET3*	AD		Hokama et al., 2014; Santos-Cortez et al., 2018

*AD, Alzheimer disease; PD, Parkinson disease; HD, Huntington’s disease; SCZ, schizophrenia; ASD, autism spectrum disorders; FXS, fragile X syndrome; PWS, Prader–Will syndrome.*

#### Monogenetic Obesity Genes

##### Leptin and leptin receptor (LEP and LEPR)

Leptin is a hormone expressed and secreted by adipose tissue (Masuzaki et al., 1995). It is a cytokine/adipokine essential for the regulation of energy balance through feeding behavior and energy expenditure (Zhang et al., 1994). Leptin is an anorexigenic hormone, stimulating the expression of anorexigenic neuropeptides (POMC and α-MSH) and inhibiting the expression of orexigenic neuropeptides (NPY and AGRP) (Jéquier, 2006). The desire to eat is reduced by leptin signals by binding to the leptin receptor in the arcuate nucleus of the hypothalamus and stimulating thermogenesis and satiety.

Leptin receptor β (LEPRβ) is expressed in the neocortex, hypothalamus, medulla, and cerebellum (Burguera et al., 2000). LEPRβ activates the Janus kinase 2 and signal transducer and activator of transcription 3 (JAK-2/STAT3) pathway (Baumann et al., 1996; Bjørbaek and Kahn, 2004; Arnoldussen et al., 2014). This signaling pathway is associated with multiple physiological and pathological regulation processes. It can induce the expression of high-mobility group box 1 (*HMGB1*), which promotes the release of cytokines as Tumor Necrosis Factor-Alpha (TNFα), inducing the inflammatory reaction (Liu et al., 2007).

Many mutations in the *LEP* and *LEPR* genes have a major influence on metabolism, leading to obesity (Ramachandrappa and Farooqi, 2011; Wasim et al., 2016). Both *LEPR*- and *LEP*-deficient individuals exhibit rapid weight gain in the first few months of life, with endocrine abnormalities and severe hyperphagia (Saeed et al., 2014).

Leptin plasma levels are highly correlated to IR, adipocyte number, and fat mass (Friedman and Halaas, 1998). However, in spite of high leptin levels during obesity, a failure in the essential leptin mechanisms (reduction in feeding behavior and increased energy expenditure) is present (Frederich et al., 1995) due to leptin resistance (Morris and Rui, 2009).

In the last few years, many studies have shown the role of leptin and leptin receptor in neurological and NDgD. *In vitro* studies showed that inhibition of the JAK/STAT3 pathway protects against α-synuclein (α-SYN), induced neuroinflammation and dopaminergic neurodegeneration (Qin et al., 2016). α-SYN is a presynaptic protein that plays a central role in the pathophysiology of PD through neuroinflammatory response (Roodveldt et al., 2008). Genetic missense mutations in α*-SYN* gene have been associated with familial forms of PD (Polymeropoulos, 1997; Singleton, 2003).

Moreover, leptin can reduce TAU phosphorylation triggering neuroprotective effects. A leptin-resistant mouse model (*Lepr db/db*) developed obesity and diabetic phenotypes showing high levels of TAU protein phosphorylation. TAU aggregation and hyperphosphorylation trigger cytotoxicity and development of NDgD. This phosphorylation increases the risk for developing dementia (Platt et al., 2016).

Recently, a study demonstrated that high-fat diet rat offspring showed cognitive damage and lower *Insr*, *Lepr* expression levels in hippocampus, persisting up to postnatal day 150. Thus, maternal exposure to high-fat diet during pregnancy and lactation can modulate cognition and behavior on adult offspring through *Lepr* (Cordner et al., 2019).

##### Proopiomelanocortin (POMC)

Proopiomelanocortin is a prohormone that suffers posttrans lational proteolysis, generating the active hormones α-, β-, and γ-, melanocyte-stimulating hormones (MSHs) and adrenocorticotropic hormone (ACTH), which all have a wide range of physiological actions (Cone, 2005; Toda et al., 2017). POMC is a key component of the melanocortin–leptin system, which regulates food intake and energy balance (Mountjoy, 2015). Then, mutations in *POMC* gene have been associated with morbid obesity (Ramachandrappa and Farooqi, 2011; Nordang et al., 2017).

In the brain, *POMC* is expressed in the arcuate nucleus (ARC) of the hypothalamus, the pituitary gland, and the brain stem (Toda et al., 2017). POMC neurons in the ARC integrate peripheral signals such as leptin, insulin, and glucose, which regulate energy balance by inducing satiety and higher energy expenditure. Satiety is mediated by the action of POMC peptides (α and β-MSH) on MC4R in the PVN of the hypothalamus (Andermann and Lowell, 2017; Toda et al., 2017; Candler et al., 2019).

The relationship of POMC with NDgD has been demonstrated with a specific AD animal model. This AD animal model (3xTg-AD) is a transgenic mouse expressing three dementia-relates genes: presenilin-1 (*PS*_1m146V_), amyloid precursor protein (*APPS*_we_), and microtubule-associated protein tau (*tau_P301L_*). This model exhibited plaque and tangle pathology, synaptic dysfunction, and showed amyloid (β and TAU pathology (Oddo et al., 2003; Sterniczuk et al., 2010). Hypothalamic gene expression showed higher mRNA expression of genes related to inflammation and apoptosis, as well as lower levels of POMC and NPY-expressing neurons. However, voluntary exercise training reduced apoptosis and increased POMC and NPY-expressing neuronal populations (Do et al., 2018). Early exercise intervention can normalize hypothalamic inflammation, neurodegeneration, and the glucose metabolism in 3xTg-AD model, suggesting that exercise can reduce the progression of dementia and AD (Do et al., 2018).

On the other hand, an HD mouse model (R6/2), which has early dysregulations in the corticostriatal pathway (Cepeda et al., 2007) and hyperactive striatal neurons (Walker et al., 2008), exhibits a reduction of the feeding-related neuropeptides (*POMC, NPY*, and *CART*) (van der Burg et al., 2008). In addition, high-fat diet induced a reduction of synaptic inputs in hypothalamic nuclei [including lateral hypothalamus (LH) and ARC] and apoptosis of NPY/AGRP and POMC neurons. This dysfunction is associated with hypothalamic neurodegeneration (Tabrizi et al., 2011; Sousa-Ferreira et al., 2014). Expression of feeding-related neuropeptides in hypothalamic progenitor cells showed that POMC, CART, and NPY could be involved in the development of HD (Sousa-Ferreira et al., 2011).

Besides POMC, another anorexic neuropeptide is the cocaine and amphetamine regulated transcript (CART). CART is a neuropeptide expressed in the brain that is involved in reducing food intake and regulating energy homeostasis (Douglass et al., 1995; Banke et al., 2013). CART was associated with the development of HD (Gabery et al., 2010). Increased *CART* levels in the cerebrospinal fluid were associated with an increased number of CART immunopositivity neurons in the hypothalamus of HD patients (Cheong et al., 2019).

In patients with PD and ischemic stroke, levels of dopamine (DA) are reduced (Calne and Sandler, 1970; Bhakta et al., 2014). Administration of exogenous DA in *ex vivo* neurons induced *CART* expression and showed protection against brain damage by reducing inflammation activation (Lin et al., 2018). All of these confirm that CART could be involved in the development of NDgD as HD and PD.

On the other hand, NPY is an orexigenic neuropeptide associated with obesity, which is related to appetite regulation and development obesity (Wu et al., 2019). The NPY system is expressed in the peripheral nervous system and in the CNS (hippocampus, basal ganglia, and brain stem (Allen et al., 1986). Some monogenetic studies have reported the association of NPY with neurodevelopment, AD, PD, and HD (Ahmed et al., 2019; Li et al., 2019).

##### Melanocortin 4 receptor (MC4R)

Melanocortin is produced from the cleavage of the POMC precursor. This protein will then bind to one of its receptors. There are five known MCRs designated as MC1R through MC5R (Gantz et al., 1993, 1994; Roselli-Rehfuss et al., 1993; Yang et al., 2000). *MC4R* is predominantly expressed in the CNS including the hypothalamus, thalamus, hippocampus, brain stem, and cortex, although it is also detected in peripheral tissues. In addition, it could be expressed by neurons, microglia, and astrocytes (Chen et al., 2018). In particular, MC4R is activated by the POMC-derivate neuropeptides (α- and β-MSH) and blocked by agouti-related protein (AgRP) expressed in AgRP/NPY neurons in the ARC. The function of these neurons is modulated by signals from adipose tissue or the gut, such as leptin, ghrelin, and NPY (Clément et al., 2018).

The MC4R signaling pathway is necessary to control the energy balance, thermogenesis, and peripheral glucose metabolism, which involves G protein-mediated activation of adenylate cyclase and augmented cAMP production (Vollbach et al., 2017). As mentioned above, mutations in the *MC4R* gene have been associated with early-onset obesity and severe hyperphagia, causing about 5% of severe obesity in children and adults (Farooqi et al., 2000; Vaisse et al., 2000; Ramachandrappa and Farooqi, 2011).

However, in the brain, MC4R is involved in anorexigenic, ant-inflammatory, and antiapoptotic effects (Caruso et al., 2013). In addition, AD transgenic mouse model 3xTg-AD showed lower levels of *MC4R* and *AgRP* mRNA compared to the control (Do et al., 2018), confirming the important role of MC4R in the development of AD.

Another study showed that MC4R activation can inhibit the overexpression of inflammatory cytokines (*IL-6*, *IL-1*, *IL-1*β, and *TNF*α) in cerebral ischemia and AD (Giuliani et al., 2011; Spaccapelo et al., 2013).

##### Preproconvertase-1 (PCSK1)

*PCSK1* is the gene that encodes the proprotein convertase 1/3 (PC1/3), which is a principal processing enzyme of precursor proteins in the secretory pathway. It is expressed in the brain, neuroendocrine system, and enteroendocrine cells (Creemers, 2008; Choquet et al., 2011). PC1/3 is synthesized as proPC1/3, which is inactive and is quickly converted into PC1/3 by autocatalytic excision of the NH2-terminal propeptide in the endoplasmic reticulum (ER). For fully PC1/3 activation, a second internal rupture of the propeptide is required in the post-ER compartment (Muller and Lindberg, 1999). An example of PC1/3 substrate is POMC, which is expressed in different neural cell populations of the ARC. In addition, PC1/3 acts in concert with PC2 to process POMC and obtain different neuropeptides as α-MSH [reviewed in Stijnen et al. (2016)].

Deficiency of *PSCK1* was associated with recessive monogenic obesity (Ramachandrappa and Farooqi, 2011). Mutations in *PSCK1* were associated with early-onset obesity, hyperphagia, sensitive hypoglycemia, and endocrine disorders (Jackson et al., 1997; Farooqi et al., 2007). However, *PCSK1*-null mice are not obese but showed growth retardation and multiple neuroendocrine abnormalities (Zhu et al., 2002; Choquet et al., 2011; Creemers et al., 2012). Moreover, PCSK1 deficiency has been associated with a major neuroendocrine disease, the Prader–Willi Syndrome (PWS). This disease is a complex genetic disorder characterized by hypogonadism, obesity, hyperphagia, growth impairment, and cognitive impairments (Ramos-Molina et al., 2018).

On the other hand, *PCSK1* expression in the hypothalamus is high in POMC and AgrP/NPY neurons, both leptin-responsive neuronal populations with ARC. PC1/3 activity is essential for pre-AGRP and POMC processing in the ARC (Ramos-Molina et al., 2016). The expression profiles in postmortem human brains showed downregulation in *PCSK1* and other 11 metabolic genes (Hokama et al., 2014). In the cortex of 3xTg-AD mouse model, *Pcsk1* expression was downregulated, which could correlate with cognitive impairment (Oddo et al., 2003; Hokama et al., 2014; Castillo et al., 2017).

##### Single-minded 1 (SIM1)

SIM1 is a member of the basic helix-loop-helix Per-Arnt-Sim (β-HLH-PAS) family of transcription factors. SIM1 is critical for the formation of the PVN in the hypothalamus in mice (Michaud et al., 1998). Homozygous *Sim1*-knockout mice (*Sim1^–/–^*) lack PVN and die perinatally. However, heterozygous *Sim1^+/–^* mice are viable, presenting an early-onset obesity, hyperphagia, and increased linear growth, similar to *Mc4r*-mutant mice (Michaud, 2001). A few mutations in *SIM1* gene have been found in obese individuals (Ramachandrappa and Farooqi, 2011), affecting the SIM1 transcriptional activity (Zegers et al., 2014).

*SIM1* gene, like other metabolic genes, participates in the development of NDgD and NDvD. Defects in the serotonergic systems are associated with depression, obsessive–compulsive disorder, and SCZ. In addition, degeneration of mesencephalic dopaminergic (mDA) neurons is associated with PD. *Sim1^–/–^* newborn mice were used to evaluate *Sim1* impact in mDA neuron differentiation and rostral 5-hydroxytryptamin (5-HT) neurons. They found a reduction in the number of dorsal raphe nucleus (DRN) 5-HT neurons, suggesting that *Sim1* may modulate serotonin release *via* regulator of G protein signaling 4 (RGS4) (Osterberg et al., 2011). The role of RGS4 in not well understood, but it can modulate 5-HT 1A-mediated neurotransmitter release *in vitro* and *in vivo* (Beyer et al., 2004; Ghavami et al., 2004). All of these suggest that *SIM1* could have an important role in the development of NDgD as PD (Osterberg et al., 2011) and NDvD as SCZ (Purcell et al., 2014).

##### Brain-derived neurotrophic factor (BDNF)

BDNF is a neurotrophic factor that plays a fundamental role in the development and plasticity of the CNS. BDNF binds to the tropomyosin-related kinase receptor (TrkB) (Di Carlo et al., 2019). BDNF is a key factor in brain signaling and synaptic plasticity (Hofer et al., 1990; Kowiañski et al., 2018). BDNF/TrkB neurotrophic signaling regulates the migration, development, differentiation, and survival of fetal neurons (Chaldakov et al., 2007), and is a major participant in the regulation of food intake (Rosas-Vargas et al., 2011). Mutations in *BDNF* gene have been associated with monogenetic obesity (Ramachandrappa and Farooqi, 2011).

Recently, numerous studies have shown the important role of BDNF in the development of NDgD and neurodevelopment. BDNF was associated with HD and AD (Couly et al., 2018; Smith-Dijak et al., 2019). Deficient BDNF/TrkB activity triggers neurodegeneration in AD *via* the activation of JAK2/STAT3 pathway and increasing inflammatory cytokines in human AD brains (Wang Z. H. et al., 2019). BDNF levels and its signaling have been modulated in the etiopathogenesis of AD, which suggested that BDNF levels could be a biomarker for AD [reviewed in Balietti et al. (2018)]. Moreover, a recent study showed that the P42 peptide treatment alleviates HD deficits in motor performance by changing BDNF level and activity (Couly et al., 2018).

As previously reported, the exposition to heavy metal can cause cognitive impairment and depressive disorders through BDNF. In early pregnancy, higher arsenic levels in blood were associated with lower levels of BDNF. The heavy metal exposure could trigger maternal depressive disorder and newborn neurodevelopment by lower levels of BDNF (Zaw and Taneepanichskul, 2019).

##### Tropomyosin-related kinase B (TrkB)

TrkB or neurotrophic receptor tyrosine kinase 2 (NTRK2) is a receptor for neurotrophin (NT) 4, BDNF, and NT-3 (Klein et al., 1990; Squinto et al., 1991). Some neurological diseases, obesity, and eating disorders have been associated with dysregulation of TrkB (Desmet and Peeper, 2006; O’Rahilly and Farooqi, 2006; Luberg et al., 2010). Mutations in the gene encoding TrkB, *NTRK2*, have been associated with monogenetic severe obesity with developmental delay (Ramachandrappa and Farooqi, 2011). These mutations modify TrkB ability to stimulate neurite outgrowth in response to BDNF. Thus, reduced hypothalamic neurogenesis could play a role in obesity and severe hyperphagia (Gray et al., 2007). The dorsomedial hypothalamus (DMH) has a role in the regulation of energy expenditure. A recent study revealed that the activation of *TrkB*-expressing DMH neurons suppresses appetite and maintain physiological satiety. It indicates that BDNF can modulate in part on the DMH to control bodyweight, suggesting that activation of DMH by the TrkB neurons could be a powerful way to treat obesity (Liao et al., 2019).

Recently, studies showed that TrkB can be involved in the development of NDgD. TrkB is widely distributed in different regions of the human brain, specifically in the dopaminergic neurons of the substantia nigra. In PD patients, the *TrkB* expression in the substantia nigra is significantly lower [reviewed in Jin (2020)], suggesting a role of this gene in the development of NDgD.

#### Polygenetic Obesity Genes

Obesity is a multifactorial and polygenic disease in which variants of different genes are associated with this disease and with the development of NDgD and NDvD (Arnoldussen et al., 2014; Lee and Mattson, 2014). As mentioned above, over 900 genetic variants have been associated with BMI and 346 *loci* were associated with body fat distribution (Yengo et al., 2018; Pulit et al., 2019). In this review, we will briefly described a few of the genes associated with obesity that have been discovered by a GWAS approach and that had been replicated in different populations. The genes reviewed here are *FTO, NRXN3, NPC1, NEGR1, MTCH2*, and *GNPDA2*, which were chosen for their association with obesity and their influence on NDgD or NDvD.

##### Fat mass and obesity-associated gene (FTO)

*FTO* gene is one of the most studied genes due to its association with obesity (Locke et al., 2015). A common variant of the *FTO* gene was identified through a T2D GWAS in 2007, and it also showed a strong association with obesity (Frayling et al., 2007; Scuteri et al., 2007). *FTO* association with obesity is the most replicated in different populations worldwide (Dina et al., 2007; Andreasen et al., 2008; Villalobos-Comparán et al., 2008).

*FTO* protein function was first described as an N^6^-methyladenosine (m^6^A) demethylase dependent of iron and 2-oxoglutarate (Gerken et al., 2007; Jia et al., 2011). Then, *FTO*-deficient mouse model was studied to understand its physiological function. These mice display postnatal growth retardation and reduced food intake, with a reduction of adipose tissue (Fischer et al., 2009; Gao et al., 2010). On the contrary, the overexpression of *FTO* showed a reduction of adipose tissue (Church et al., 2010). However, it took 8 years to understand that *FTO* intronic variant associated with obesity does not regulate *FTO* expression. In fact, this variant disrupts the binding site of the ARID5B repressor, which regulates *IRX3* and *IRX5* expression, genes involved in adipogenesis, thermogenesis, and lipid accumulation (Claussnitzer et al., 2015). This is an example that the discovery of new genetic variants could unveil an unexpected impact on physiology.

Over the last few years, *FTO* was associated with NDgD, especially AD. Obesity and T2D in human and mice can activate *FTO* in the brain tissues by defective insulin signaling (Li et al., 2018). It is known that obesity and T2D are commonly associated with the development of AD (Vagelatos and Eslick, 2013). A recent study demonstrated that in the 3xTg-AD mouse model, *Fto* neuronal conditional silencing reduced the cognitive deficits, suggesting its implication on the insulin signaling defect present in AD (Li et al., 2018).

##### Neurexin 3 (NRXN3)

NRXN3 is a type of neurexins, which are neuron-specific cell surface proteins. Their structure suggests a role in the cell adhesion and cell recognition (Ushkaryov et al., 1992).

*NRXN3* gene has been associated with waist circumference as an obesity trait (Heard-Costa et al., 2009). Moreover, this gene was also associated with increased BMI and visceral fat and decreased sleep duration (Prats-Puig et al., 2013).

*NRXN3* is involved in synaptic function in the cognitive decline associated with aging and AD. Moreover, mutations in *NRXN3* have been identified in AD patients (Zheng et al., 2018; Hishimoto et al., 2019), and rare deletions have been associated with ASD (Vaags et al., 2012).

##### Niemann–pick type C1 (NPC1)

NPC1 is a protein that regulates the transport of cholesterol and fatty acids from late endosomes/lysosomes to cellular structures as mitochondria and plasma membrane for maintaining lipid homeostasis [reviewed in King and Sharom (2012)]. In humans, GWAS showed common *NPC1* variants associated with obesity (Meyre et al., 2009).

A rare autosomal recessive mutation in human *NPC1* causes a disorder in lipid storage leading to progressive and lethal neurodegeneration and lung and liver failure [reviewed in Lamri et al. (2018)]. NPC disease is a lysosomal storage disorder, present in childhood with accumulation of visceral lipid and progressive neurodegenration, with characteristic dysphagia, cerebellar ataxia, and dementia resulting in a lower life expectancy (Newton et al., 2018). *NPC1* gene has been associated with NDv and NDgD as AD (Rouillard et al., 2016) and SCZ (Kawazoe et al., 2018).

##### Neuronal growth regulator 1 (NEGR1)

*NEGR1* is expressed in the rat brain. It has been proposed that NEGR1 could modulate the intracellular cholesterol trafficking, suggesting its implication in human obesity (Kim et al., 2017). *NEGR1* was associated with obesity (Thorleifsson et al., 2009; Willer et al., 2009). Moreover, *Negr1*-deficient mice showed increased adiposity (Joo et al., 2019). The genetic variability in *NEGR1* could be associated with psychological traits of patients with eating disorders, like bulimia (Gamero-Villarroel et al., 2015).

Moreover, a GWAS reported a *NEGR1* variant associated with major depression (Wray et al., 2018). Recent studies on humans and animals support the idea that NEGR1 is involved in psychiatric disorders such as SCZ (Karis et al., 2018), ASD (Szczurkowska et al., 2018), and AD (Ni et al., 2018; Raghavan et al., 2019).

*Negr1* deficiency in animals leads to impaired cortical development and impaired behavior, a conduct similarly observed in ASD patients (Szczurkowska et al., 2018). On the other hand, SCZ patients showed higher levels of *Negr1* in the dorsolateral prefrontal cortex (Karis et al., 2018). Therefore, these data demonstrate the implication of *NEGR1* in NDvD.

##### Mitochondrial carrier homolog 2 (MTCH2)

MTCH2 is a relative novel protein located in the inner membrane of mitochondria. It is expressed in white adipose tissue. MTCH2 has an important regulatory role in the differentiation and biology of the adipocyte (Bernhard et al., 2013). *MTCH2* variants have been associated with increased BMI, obesity, and diabetes (Willer et al., 2009; Heid et al., 2010). A study on *Mtch2-*knockout mouse model reported that the animals die at an embryonic 7.5 day, suggesting that *Mtch2* could play a specific role in embryonic development (Ruggiero et al., 2017).

It is relevant mentioning that the lower expression of *MTCH2* was associated with late onset AD status in a GWAS (Karch et al., 2016). A case control study in Swedish population showed several variants associated with SCZ by exome-sequencing (Purcell et al., 2014). These data suggest the possible role that *MTCH2* could have in the development of NDgD and NDvD.

##### Glucosamine 6 phosphate isomerase 2 (GNPDA2)

*GNPDA2* gene encodes the enzyme glucosamine-6-phosphate deaminase (GlcN6P). This enzyme catalyzes the reversible reaction of D-glucosamine-6-phosphate into D-fructose-6-phosphate and ammonium (Arreola et al., 2003). This enzyme has a hydrolase activity and is involved in metabolic pathways, glucose and nucleotide metabolism. *GNPDA2* is highly expressed by the brain (cortex and hypothalamus) (Willer et al., 2009).

*GNPDA2* gene variant was associated with obesity in populations with European ancestry (Willer et al., 2009), and this association was replicated in other populations (Renström et al., 2009; Locke et al., 2015). In a diet-induced obesity model, high-fat diet led to a lower *GNPDA2* hypothalamic expression compared to rats fed with chow diet (Gutierrez-Aguilar et al., 2012).

*GNPDA2* was overexpressed in PD and AD cases and showed lower serum levels in PD patients. Interestingly, in PD patients, GNPDA2 showed an inverse correlation with α-synulein protein levels in the cerebrospinal fluid (Lachén-Montes et al., 2019), suggesting its implication in developing PD.

### Genes Associated With Neurodegenerative and Neurodevelopmental Diseases

Genetic studies have shown genes implicated in developing NDgD and NDvD (Saad et al., 2011; Arnoldussen et al., 2014; Henriksen et al., 2017; Wu and Pan, 2018).

The heritability for AD is between 60 and 80% (Wingo, 2012). AD genetics has been reviewed elsewhere (Reitz, 2015; Goldman and Van Deerlin, 2018; Jung et al., 2018). However, only 1% of all AD is autosomal dominant genes, such as presenilin 1 (*PSEN1*), presenilin 2 (*PSEN2*), and amyloid precursor protein (*APP*) (Hinz and Geschwind, 2017).

With the genomic era, several GWAS and meta-analysis have been performed, identifying up to 29 risk *loci* associated with AD, and *APOE* is one of them (Lambert et al., 2013; Jansen et al., 2019).

Interestingly, some genes associated with obesity were also associated with AD by different genetic approaches: candidate gene approach *FTO* (Li et al., 2018), by differentially expressed genes *NRXN3* (Zheng et al., 2018; Hishimoto et al., 2019), *NPC1* (Rouillard et al., 2016), *NEGR1* (Wray et al., 2018), or by GWAS approach: *LEPR*, *BDNF*, *TNF*α, and *MTCH2* (Gao et al., 2015; Karch et al., 2016; Lemche, 2018).

In respect to PD, this disease only affects 2% of the population over 60 years old. Family-based genetic studies had identified 23 genes associated with PD, having diverse functions as deficiency of synaptic transmission, vesicular recycling, lysosomal dysfunction, mitophagy. Some of those genes are: *synuclein-Alpha* (*SNCA*), inherited in an autosomal dominant; *PARKIN*, autosomal recessive juvenile parkinsonism; PTEN-induced kinase (*PINK1*), recessive early onset, among others (Karimi-Moghadam et al., 2018).

Meta-analysis from PD GWAS report over 30 variants close to genes like *CTSB*, *TMEM175*, *LRRK2*, among others (Nalls et al., 2014; Chang et al., 2018). Some of the common genes that have been associated with obesity and PD are: *GNPDA2* (Lachén-Montes et al., 2019) *CD38*, *TNFα*, and *PAI-1* (Pan et al., 2018) that will be discussed below.

Huntington’s disease is another NDgD characterized by progressive neurodegeneration with severe neuronal loss (90%) that occurs in the lateral hypothalamus (Sprengelmeyer et al., 2006), as well as neuronal degeneration and hypothalamic dysfunction (van Duijn et al., 2014). In this disease, the gene *HTT*, encoding for the huntingtin protein, has a CAG trinucleotide expansion mutation, causing behavioral abnormalities, dementia, and progressive movement disorder (Ross et al., 2014). A GWAS reported a few gene variants associated with HD that are close to the *MLH1, FAN1, MTRM10, RRM2B*, and *UBR5* genes. Some of these genes are implicated in DNA mismatch repair, structure-specific DNA handling, mitochondrial energetics, and oxidative stress (Lee et al., 2015). However, genes associated with obesity have been also described to be associated with HD, such as *POMC*, *NPY*, *CART*, and *BDNF* (Sousa-Ferreira et al., 2011).

Neurodevelopmental diseases are a group of early-onset neurological disorders, which also have a genetic background. These disorders include ASD that often present intellectual disability (ID), motor abnormalities, and epilepsy. Fragile X syndrome (FXS) is part of the syndromic ASD classification (Tãrlungeanu and Novarino, 2018).

Fragile X syndrome is an inherited disease linked to the X chromosome and is one cause of intellectual disability. This syndrome is associated with a triplet repeats of cytosine-guanine-guanine (CGG) in the *FMR1* gene (O’Donnell and Warren, 2002; Visootsak et al., 2014). Usually, FXS patients have social anxiety, severe communication deficits, and stereotyped behavior (Hall et al., 2009). Some cases with *FMR1* premutation showed lack of satiation, severe hyperphagia, and severe obesity (Martínez-Cerdeño et al., 2017).

Schizophrenia is another NDvD that affects 1% of the worldwide population. It is a chronic and severe mental disorder, characterized by cognitive impairment. More than 1,000 candidate genes have been proposed to be associated with SCZ. However, their functional implication in developing SCZ remains unclear. Hu et al. (2013) analyzed the new genetic variants associated with SCZ and identified pathways that might explain the biological function causing this disease. They propose that pathways involved in fatty acid degradation, glycan degradation, PPAR signaling, among others, could be involved in the development of this disease. However, the genes that have been associated with SCZ are *NRXN3*, and *NEGR1, NPC1, and TNF*α (Hu et al., 2013; Park et al., 2019).

Frequently, the associations between genes and diseases are studied separately. Interestingly, a recent genetic study analyzed the association of millions of genetic variants with BMI and major psychiatric disorders (SCZ, bipolar disorder, and major depression), instead of analyzing separately each disease. This study identified 111 genetic loci overlapping between BMI and the psychiatric disorders. Some of these variants are associated with genes expressed in the brain with plausible functions in CNS development, intracellular processes, and GABAergic and glutamatergic signaling (Bahrami et al., 2020); however, their functions have to be confirmed.

In the next section, we will briefly describe genes associated with NDgD as AD, PD, and HD that were discovered by a GWAS approach and meta-analysis (Qi et al., 2012b; Moss et al., 2017; Chang et al., 2018; Jansen et al., 2019) and that had previously shown strong association with monogenic or polygenic obesity (Tong, 2011; Hatziri et al., 2018; Wang et al., 2018a, b; Liu et al., 2019). Some of the genes mentioned below are *APOE* and *TREM2* associated with AD; *CD38* variants with AD and PD; and *SYT4* with PD (Chang et al., 2018; Jansen et al., 2019). In addition, genetic studies found the association of SIRT1 with PD and AD (Zhang A. et al., 2012; Rana et al., 2019) and with SCZ (Kishi et al., 2011). A similar strategy to select the genes associated with NDvD as SCZ and ASD. However, there are just a few genes highly associated with the development of NDvD and obesity. Some of them were discovered by exome-sequencing studies as *MTCH2* and *SIM1*, which were associated with the development of SCZ (Purcell et al., 2014). GWAS showed that *FMR1* was associated with ASD and FXS (Tassone et al., 2000; Tassone et al., 2007) and *TET3* gene variants with the development of NDvD (Santos-Cortez et al., 2018). However, little is known about the function of these genes and their implications in NDgD and NDvD.

#### Apolipoprotein E (APOE)

Apolipoprotein E is a glycoprotein that is produced predominantly by astrocytes in the brain and peripherally in the liver (Huang and Mahley, 2014). There are three *APOE* alleles: E2, E3, and E4. Peripherally, APOE2 and APOE3 bind to high-density lipoproteins (HDLs), responsible for trafficking lipids from the periphery cells to the liver for elimination. APOE4 have greater affinity for very low-density lipoprotein, and it is less efficient at homeostatic maintenance (Huang and Mahley, 2014). However, *APOE4* was associated with higher fasting glucose and insulin levels, as well as an increased metabolic syndrome risk with younger age onset (Torres-Perez et al., 2016). In obese men, *APOE4* carriers have elevated levels of plasma cholesterol, triglycerides, glucose, and insulin and presents IR (Elosua et al., 2003; Jones and Rebeck, 2019).

The most common *APOE3* allele has been associated with an average risk to develop AD. However, *APOE*4 homozygotes increase the risk 15 times to the development of AD. In addition, *APOE4* is also associated with increased risk for CVD and metabolic syndrome (El-Lebedy et al., 2016).

Interestingly, *APOE* is one of the metabolic genes associated with the development of NDgD. *APOE4* has shown a strongest association with the development of late-onset AD (Lambert et al., 2013; Jansen et al., 2019). APOE affects TAU pathogenesis, neuroinflammation, and TAU-mediated neurodegeneration (Yoshiyama et al., 2007). *APOE4* carrier has increased Aβ accumulation and decreased clearance in AD brains (Liu et al., 2013; Shi et al., 2017).

#### Cluster of Differentiation 38 (CD38)

CD38 is a type II transmembrane glycoprotein (Jackson and Bell, 1990). It is a lymphocyte-specific antigen, has ectoenzymatic activity, and functions as a receptor and adhesion molecule (States et al., 1992). *CD38* is highly expressed on plasma cells, red blood cells, platelets (Deaglio et al., 2001), and adipose tissue and in obese people (Nair et al., 2005; Mutch et al., 2009). CD38 is involved in different biological processes including cell proliferation, hormone secretion, muscle contraction, egg fertilization, and immune response. It is also involved in the catabolism of nicotinamide adenine dinucleotide (NAD^+^) and nicotinamide adenine dinucleotide phosphate (NADP) (Howard et al., 1993).

A *CD38*-deficient mouse model has higher metabolic rate and showed protection against diet-induced obesity through increasing NAD-dependent activation of sirtuin (SIRT) proliferator-activated receptor gamma coactivator 1-alpha (PGC1α), which is involved in the regulation of mitochondrial biogenesis and energy homeostasis (Barbosa et al., 2007). On the other hand, a recent study showed that CD38 participates in adipogenesis and lipogenesis of adipose tissues through regulating Sirt1-mediated signaling pathway (Wang et al., 2018b).

In addition to its role in obesity, *CD38* is also expressed in neurons, microglial cells, and astrocytes [reviewed in Guerreiro et al. (2020)]. *CD38* is highly expressed in the mouse brain during development (postnatal days 14 and 28). The *CD38*-knockout mice showed that the brain displayed a 10-fold NAD^+^ level more than wild type, suggesting that CD38 is one regulator of intracellular NAD^+^ levels in the brain.

Genomic studies demonstrated that *CD38* was associated with PD (Saad et al., 2011) and was confirmed by a meta-analysis (Chang et al., 2018). It suggests that *CD38* could play an important role in the development of NDgD as PD.

#### Sirtuin1 (SIRT1)

SIRT1 is a deacetylase NAD^+^-dependent that is expressed in the heart, adipose tissue, muscle, kidney, liver, and brain (basal ganglia, prefrontal cortex, and hippocampus, which are areas associated with NDgD) (Zakhary et al., 2010). SIRT1 is involved in important processes such as cell cycle regulation, energy metabolism modulation, mitochondrial biogenesis, glucose/cholesterol metabolism, etc. (Herranz and Serrano, 2010; Nogueiras et al., 2012). SIRT1 has been associated with obesity (Zillikens et al., 2009) and is involved in food intake regulation, life span, diabetes, and CVD (Pfluger et al., 2008; Nogueiras et al., 2012).

*SIRT1* expression and activity were increased by resveratrol treatment, protecting neuronal cells (Seo et al., 2012; Herskovits and Guarente, 2014) and accelerating brain aging (Duan, 2013). SIRT1 has shown an important role in the regulation of other anti-aging genes as *KL* (*Kloto*), *SHC1 or p66Shc* (*transforming protein SCH1*), and *FOXO1a/FOXO 3a* (Forkhead box) by p53 transcription factor deacetylation (Seo et al., 2012; Herskovits and Guarente, 2014). Low expression of SIRT1 in adipose tissue was shown in obese subjects (Stefanowicz et al., 2018).

Moreover, low levels of *SIRT1* are present in NDgD as AD and PD (Lutz et al., 2014; Singh et al., 2017). In addition, genetic studies found the association of *SIRT1* with PD and AD (Zhang A. et al., 2012; Rana et al., 2019) and with SCZ (Kishi et al., 2011).

#### Tumor Necrosis Factor-Alpha (TNFα)

Tumor necrosis factor-alpha is a potent pleiotropic pro-inflammatory cytokine (Hajeer and Hutchinson, 2000, 2001). TNFα is produced by many cell types such as neutrophils, fibroblasts, keratinocytes, macrophages, natural killer cells, T and B cells, and tumor cells (Anderson et al., 2004). TNFα plays an essential role in chronic inflammation associated with different pathologies, such as obesity, T2D, AD, and PD (Wei et al., 2011).

Tumor necrosis factor-alpha has been associated with NDgD and NDvD in many reports (Arnoldussen et al., 2014). The neuroinflammation in the NDgD, such as AD, PD, amyotrophic lateral sclerosis, and multiple sclerosis is modulated by cytokines like TNFα (Baj and Seth, 2018). Higher levels of cytokines as TNFα, IL-10, TNFβ, and CRP were associated with NDvD, as SCZ and ASD (Bodnar et al., 2018).

#### Plasminogen Activator Inhibitor (PAI-1)

Plasminogen activator inhibitor is a single-chain glycoprotein belonging to the serine protease inhibitor (serpin) superfamily. There are four PAIs: PAIs-1 to 3 and protease nexins (Feinbloom and Bauer, 2005). PAI-1 inhibits the plasminogen activator and is produced by platelet, hepatocytes, adipocyte, vascular smooth muscle cell, fibroblast, and macrophages (Placencio and DeClerck, 2015). *PAI-1* mRNA expression in visceral and subcutaneous adipose tissue was correlated with BMI and severe obesity (Alessi et al., 2000). Plasma PAI-1 activity and antigen were positively associated with BMI in hypertense men (Skurk et al., 2004). The expression of *PAI-1* can be regulated by lipid/glucose metabolites, environmental factors, inflammation, age, BMI, and lifestyle. Also, chemical messengers like TNFα, hormones, inflammatory cytokines, growth factors, and endotoxins can modulate its expression (Oishi, 2009).

Recently, a new study showed the association of higher levels of PAI-1 with PD (Pan et al., 2018). *PAI-1* polymorphisms can change focal and brain stem neurological signs in patients with traumatic brain injury (Pan et al., 2018). These results suggested that *PAI-1* can participate in the development of NDgD (Arnoldussen et al., 2014).

#### Trigger Receptor Expressed on Myeloid Cells 2 (TREM2)

*TREM2* is expressed on myeloid cells (macrophages, dendritic cells, and microglia) (Bouchon et al., 2001; Daws et al., 2001; Wang et al., 2015; Ulland et al., 2017) and in adipose tissue (Park et al., 2015). TREM2 regulates the behaviors of different cell biologicals: survival, proliferation, differentiation, phagocytosis, and inflammatory response (Zhong et al., 2015; Kober and Brett, 2017). It also acts as a lipid sensing receptor to recognize and bind lipids (Wang et al., 2015).

*TREM2* gene expression was upregulated in adipose tissue in obesity animal models (Fujimoto et al., 2011; Grant et al., 2011; Park et al., 2015). *Trem2*^–/–^ mice fed with a high-fat diet showed lower mass but higher hypertrophy in adipocytes and increased adipocyte death. In addition, these mice had deficient inflammatory response of adipose tissue macrophages and severe hepatic steatosis. They showed that the function of TREM2 is a feedback mechanism to control obesity-induced IR by regulating adipose tissue remodeling pathways (Liu et al., 2019).

Furthermore, *TREM2* genetic variants have been associated with a higher risk to develop AD. The amyloid plaque compaction depends on TREM2 mechanism, forming a protective barrier that attenuates toxicity nearby neurons [reviewed in Yeh et al. (2017)].

#### Synaptotagmin-4 (SYT4)

SYT4 is insensitive to Ca^2+^. *SYT4* is expressed in brain and neuroendocrine system and has been suggested to have a neuroendocrine role (Tong, 2011; Zhang et al., 2011). The upregulation of *SYT4* inhibits the release of oxytocin, which is a characteristic in obese phenotype (Tong, 2011; Zhang et al., 2011). The negative regulation of *SYT4* could potentially repair or reduce the degree of diabetes neuropathies (Rahimi et al., 2015); however, the physiological function of SYT4 remains unknown.

A specific mouse model showed that *SYT4* upregulation, within dystrophic neurons, could reflect impaired protein degradation that happens in AD (Zhang et al., 2009), suggesting its implication in this disease.

#### Fragile X Mental Retardation 1 (FMR1)

*FMR1* gene encodes the Fragile X mental retardation protein (FMRP). FMR1 is found in most adult and fetal tissues, especially in brain and testes (Berry-Kravis et al., 2002; Berry-Kravis et al., 2011). It has been reported that FXS patients have obesity (Martínez-Cerdeño et al., 2017).

Trinucleotide (CGG) repeat in *FMR1* promotor region is associated with FXS, and a permutation is associated with fragile X-associated tremor/ataxia syndrome (FXSAS). Many individuals with permutation on the *FMR1* gene have high levels of mRNA, but normal FMRP is synthesized (Tassone et al., 2000, 2007). *Fxr1^–/–^*-knockout mice die at 24 h of birth, and heterozygous mice exhibit abnormal limb musculature and learning and circadian rhythm deficits (Berry-Kravis et al., 2011; Francis et al., 2014). Analysis of graph diffusion and multitask clustering of FMR1 Clip-seq and transcriptional targets showed pathways regulated by FMR1 in human neural development (Li et al., 2020).

As we previously mentioned, the function or pathway of many of the genes discovered by GWAS is unknown. Such is the case of *FMR1* with its implication in developing obesity or NDvD.

#### Ten Eleven Translocate 3 (TET3)

TET3 is a protein which belongs to the TET family, which catalyzes hydroxylation of 5-metylcytosine (5mC) to 5-hidroxymetylcytosine (5hmC) (Ito et al., 2010; Pastor et al., 2013). This is a key step in active DNA demethylation, which needs α-ketoglutarate (αKG) as a cofactor (Tahiliani et al., 2009). However, in maternal obesity, placental *TET3* methylation is increased, without totally understanding its role in obesity (Mitsuya et al., 2017).

TET3 is the most highly expressed enzyme in the brain and is an essential enzyme in neuronal differentiation and *in vivo* early neocortical development (Lv et al., 2014; Li et al., 2015). In fact,*Tet3*-knockout mouse model showed defects in brain morphology, behavior, and motor development (Santos-Cortez et al., 2018).

Interestingly, some genomic studies have shown the association of *TET3* gene variants with the development of NDvD (Santos-Cortez et al., 2018). Moreover, *TET3* expression levels are downregulated in postmortem brains of AD individuals (Hokama et al., 2014). Therefore, *TET3* is one of the new genes discovered to be associated with both NDgD/NDvD, and its mechanism leading to those diseases would have to be unveiled.

## Discussion

In this review, we addressed the obesogenic environmental conditions that influence the development of NDgD or NDvD. Interestingly, some of the environmental factors that lead to the development of obesity, NDgD, or NDvD are common. Some of the environmental factors are physical activity, alcohol consumption, socioeconomic status, parent feeding behavior, and diet. All of these factors influence the development of obesity and impair abilities as memory, fine motor skills, and cognitive function.

Obesity is a comorbidity of NDgD and NDvD due to the common biological mechanisms that affect brain cell death or brain development. The adipose tissue hypertrophy provokes inflammation, impaired insulin signaling. The secretion of adipokines and pro-inflammatory cytokines is altered, as well as the lipid metabolism. High levels of free fatty acids contribute to IR and hyperglycemia and lead to impaired cognitive processes. Moreover, oxidative damage, energy metabolism failures, mitochondrial and neurotransmission systems dysfunction could lead to cell death and NDgD. On the other hand, maternal obesity produces high levels of inflammatory mediators that can cross the blood–placenta barrier, influencing fetal development. This fetal exposure could end in neurodevelopmental disorders like SCZ, ADHD, and ASD.

Genetics plays an important role in developing obesity, NDgD, and NDvD. We described the genes associated with obesity, either on monogenic or polygenic manner. For the monogenetic obesity, several of the genes are involved in the leptin–melanocortin pathway, mainly regulated in the hypothalamus. This pathway controls the energy homeostasis, given by the food intake and the energy expenditure. Surprisingly, all these genes were also associated with NDgD or NDvD by sharing regulation on specific brain regions (hypothalamus, hippocampus, thalamus, cortex, etc.) or by leading to synaptic dysfunction and amyloid β and TAU pathology.

Moreover, we described the new genes associated with obesity, NDgD, or NDvD, as discovered by a GWAS approach. This approach has helped to discover new genes without a previous established hypothesis, thus without knowledge limitations. These genomic studies had discovered gene variants with unknown functions that we might have never imagined to be implicated in a particular disease.

In this review, we mentioned just a few genes associated with obesity, NDgD, or NDvD, but a long list of genes are associated. However, the challenge now is to identify the function of all these genes and their plausible implication in these diseases, as well as the gene–environment interaction.

In fact, gene–environment interactions have explained part of the missing heritability, but more research is needed to better understand how the genes confer susceptibility to develop a disease in the presence of an environment that enhances the trait. Therefore, our genes confer susceptibility to develop obesity, NDgD, and NDvD; however, we can modify the environment to reduce the risk.

The prevalence of obesity has risen dramatically in the last decades. As obesity is a trigger for the development of many complex diseases, as NDgD and NDvD, it is plausible that in the next decades, the prevalence of these diseases will increase. Even though we cannot fully understand the etiology of obesity, many of the risk factors for the development of obesity are already known. Then, we can modify the environmental factors to prevent obesity and avoid the risk of developing NDgD and NDvD.

## Conclusion

Obesity and NDgD/NDvD are diseases that share environmental and genetic factors, which lead to the development of all these diseases. Understanding the environment, the genes, and the gene–environment interaction involved in obesity and NDgD/NDvD, we will comprehend the etiology of these diseases. Then, by modifying our environmental factors and knowing the genetic susceptibility, we might be able to avoid the development of these diseases.

## Author Contributions

MF-D analyzed, discussed, and wrote the genetic section. YD-L searched for the bibliography and analyzed the literature. RG-A conceived, analyzed, discussed, wrote, and edited all the manuscript. All authors revised and approved the last version of this manuscript.

## Conflict of Interest

The authors declare that the research was conducted in the absence of any commercial or financial relationships that could be construed as a potential conflict of interest.
